# Estimating the opportunity cost of seasonal malaria chemoprevention implementation in Burkina Faso, Mali and Senegal

**DOI:** 10.1136/bmjgh-2024-018042

**Published:** 2025-10-03

**Authors:** Richmond Owusu, Colin Gilmartin, Halimatou Diawara, Fadima Bocoum, Oumy Ndiaye, Anika Ruisch, Genevieve Cecilia Aryeetey, Darlene Jainie, Monica Kokovena, Damian Walker, Justice Nonvignon

**Affiliations:** 1 School of Public Health, University of Ghana, Accra, Ghana; 2 Management Sciences for Health Medford, Medford, Massachusetts, USA; 3 Universite des Sciences des Techniques et des Technologies de Bamako Malaria Research and Training Center, Bamako, Mali; 4 IRSS, Institut de Recherche en Sciences de la Santé, Ouagadougou, Burkina Faso; 5 Faculty of Economics and Management, University Cheikh Anta Diop, Dakar, Senegal

**Keywords:** Malaria, Child health, Chemoprophylaxis, Control strategies, Health economics

## Abstract

Seasonal malaria chemoprevention (SMC) is a widely implemented malaria prevention strategy for children under five in the Sahel and sub-Sahel regions of Africa. This study aimed to estimate the full opportunity costs associated with SMC implementation in Mali, Burkina Faso and Senegal, addressing a gap in existing research that often focused solely on health system costs.

Using a repeated descriptive cross-sectional design, data were collected from April to November 2022 during two SMC cycles. The study involved 376 caregivers in Mali, 398 in Senegal and 373 in Burkina Faso, alongside 127 community health volunteers in Mali, 41 in Senegal and 97 in Burkina Faso. Health worker supervisors recruited were 96 in Mali, 96 in Senegal and 42 in Burkina Faso. Data collection occurred across 15 health facilities in Burkina Faso and Senegal and 16 health facilities in Mali across four districts within each country.

Both financial and economic costs were analysed using an ingredients approach, encompassing direct and indirect costs. Financial costs per SMC dose were estimated at US$0.99 in Mali, US$1.42 in Burkina Faso and US$1.51 in Senegal. Economic costs per dose were US$3.02 in Senegal, US$3.14 in Burkina Faso and US$2.96 in Mali. Total annual costs per child receiving four doses ranged from US$3.97 to US$6.05 for financial costs and US$11.85 to US$12.57 for economic costs. Notably, indirect costs, mainly related to productivity losses among caregivers, volunteers and healthcare workers, constituted 50%–66% of total economic costs.

The findings highlight the economic cost of SMC implementation, driven largely by productivity losses of caregivers and volunteers which have often been overlooked in policy decisions. This study highlights the need for comprehensive cost assessments in malaria control programmes to inform effective decision-making.

**WHAT IS ALREADY KNOWN ON THIS TOPIC**

- Economic costs incurred by government, health system and sometimes volunteers for seasonal malaria chemoprevention (SMC) implementation are known.

**WHAT THIS STUDY ADDS**

- This study extends the cost analysis to cover the productivity losses incurred by volunteers (distributors) and caregivers of children who receive SMC.

**HOW THIS STUDY MIGHT AFFECT RESEARCH, PRACTICE OR POLICY**

- The study has revealed the need to capture productivity losses/resource contribution from volunteers and caregivers in future studies.
- Policy makers will now consider the full opportunity cost of resources and not just financial cost in malaria control programmes to make informed decisions about alternative interventions.

## Introduction

In 2012, the WHO recommended administration of seasonal malaria chemoprevention (SMC) for children 3–59 months in areas with seasonal malaria transmission.^1^ In a revised guideline by the WHO, the population targeted with SMC was extended to all children belonging to age groups at high risk of severe malaria.^2^ SMC is implemented in Africa where an estimated 80% of the malaria burden in children is reported to occur.^3^ In 2023, an estimated 53 million children were reached in countries in the Sahel and sub-Sahel regions in Africa with SMC.^4 5^ The current oral formulation of SMC—comprised of sulfadoxine-pyrimethamine plus amodiaquine (SP+AQ)—is administered orally to eligible children monthly (three doses per month).^2^ In 2021, a total of 19 countries with seasonal malaria transmission implemented SMC at scale.^4^ In 19 countries of the Sahel and other seasonal areas of sub-Saharan Africa implementing SMC, approximately 221 million treatment doses of SMC were administered in 2023.^4^

Studies have reported the effectiveness of SMC at reducing the burden of malaria incidence in children.^6–12^ The most recent systematic review evidence shows that SMC resulted in substantial reduction in uncomplicated malaria incidence during the transmission season (rate ratio: 0.27, 95% CI 0.25 to 0.29 among children <5 years; rate ratio: 0.27, 95% CI 0.25 to 0.30 among children ≥5 years). Also, in high-transmission zones, SMC resulted in a moderately reduced risk of any anaemia (risk ratio: 0.77, 95% CI 0.72 to 0.83 among children <5 years).^5^ For children <10 years, there was a moderate reduction in severe malaria (risk ratio: 0.53, 95% CI 0.37 to 0.76), even though there was no evidence of reduction in malaria deaths.^5^ SMC is expected to be cost-effective in regions where malaria incidence without SMC exceeds 0.1 per child during a 4-month high transmission season. However, the annual SMC rounds should consist of between three and five cycles^13^ based on contextual factors.

While SMC has been effective in reducing the malaria burden among vulnerable children in Africa, SMC geographical coverage gaps remain.^14^ A recent systematic review found that out of the six costing studies on SMC,^12 15–18^ only one study examined the full opportunity cost of SMC implementation from a societal perspective.^19^ All the other studies partially assessed cost, particularly leaving out cost to volunteers and caregivers, and sometimes excluding capital cost. To date, many of these studies have assessed the costs of SMC implementation pilots, and not at-scale SMC programmes. Thus, costing studies of at-scale, mature SMC interventions are necessary to generate relevant evidence to guide resource allocation.

This study sought to estimate the full opportunity cost of SMC implementation in Mali, Burkina Faso and Senegal from a societal perspective. This study is different from existing studies in two main ways. Foremost, this study collected repeated cross-sectional cost data from both health system and community (household and community volunteer) perspectives, which makes the cost analysis of SMC comprehensive enough to guide priority setting and financial planning across the three countries. Having a full understanding of the resource requirements and costs of these malaria preventive interventions can facilitate evidence-informed decisions regarding the implementation or scale-up of these preventive interventions. Moreover, a comprehensive understanding of the full economic cost of implementing SMC can inform effective planning and resource allocation. For instance, the most recent and updated Malaria Guidelines by WHO have removed geographical restrictions on SMC implementation.^2^ In addition, the second edition of the field guide states that SMC should be given during peak malaria transmission season, between three and five cycles.^13^ ^5^ Furthermore, the new guideline is more flexible in recognising age-based risk among children, recommending SMC for age groups at high risk of severe malaria.^20–22^ These developments have financial and economic implications for governments and funders. Moreover, the full opportunity cost of SMC implementation will make decision makers understand how to choose between alternative malaria prevention interventions such as seasonal injectables and malaria vaccines. Finally, the results can also help in effectively planning and advocating for the allocation of sufficient financial resources and guiding the future price setting for the seasonal injectables and facilitating its implementation and scale-up in the region.

## Methods

### Study design

This study was a descriptive cross-sectional economic analysis, which collected cost data for two cycles (first and third) during the four cycles of the SMC campaigns in Senegal and five cycles in Mali and Burkina Faso in some selected districts. The cycles for SMC administration differed from one country to another; but overall, SMC administration was done between April 2022 and November 2022 across the three countries.

### SMC campaign

This study was conducted during the SMC campaign and the time horizon of the analysis was 1 year (2022). SMC administration was done on a monthly cycle basis for a minimum of four cycles in each country. However, in Burkina Faso and Mali, five cycles of SMC administration were implemented in selected districts. Each distribution period lasted 3 days (with an additional day for mop up) during which SP+AQ were administered to eligible children. In all three countries, SMC administration was done according to each country’s season of malaria transmission: July to November in Burkina Faso, April to July in Senegal and April to August in Mali. During each monthly cycle, trained SMC distributors or volunteers reached out to eligible children through door-to-door campaigns.

### Study population

The study population included healthcare workers who were supervisors, community volunteers involved in SMC implementation and caregivers of children who receive SMC.

### Sampling method

Sampling of districts and health facilities was done using the Sample Design Optimizer (SDO) tool developed by the EPIC project at the Harvard T.H. Chan School of Public Health.^23^ The following parameters were used in the SDO for determination of sample sizes: data collection unit cost, data collection budget, prior estimates of outcome of interest and sample frame. A total of 16 health facilities were selected from each country (one health facility was dropped in Burkina Faso and Senegal because of security issues) across four districts in two regions in each country (Mali—Diema, Koulikoro, Yanfolilia and Segou; Burkina Faso—Boromo, Koudougou, Reo and Diebougou; Senegal—Diourbel, Kedougou, Tamba and Kolda). Furthermore, four health facilities were randomly selected from each district. Subdistrict supervisors, mainly health workers in the subdistrict, then identified the volunteers they supervised, and these volunteers were interviewed. Subsequently, each volunteer facilitated the interview of caregivers by the research team. Overall, there were a total of 376 caregivers in Mali, 398 caregivers in Senegal and 373 caregivers in Burkina Faso. Health workers recruited were 96 in Mali, 96 in Senegal and 42 in Burkina Faso. Volunteers recruited were 127 in Mali, 41 in Senegal and 97 in Burkina Faso.

### Data collection

Standard structured costing questionnaire designed for data collection capturing all ingredients used for the SMC implementation was adapted from two previous studies.^17 24^ Using an ingredients-based approach, the study identified, measured and valued each health system and societal resource for the administration of SMC based on standard costing guidelines and practice. Costs were assigned to a matrix of line items (labour, drug, transport, infrastructure, etc.) and programmatic activity (delivery services, surveillance, pharmacovigilance, data capture, training, etc.) and evaluated for each stage of administrative layer (central, regional, district, facility/outpost). The study used Research Electric Data Capture (REDCap) software for electronic data capture in Mali and Senegal. REDCap is a free, secure, web-based application designed to support data capture for research studies. In Burkina Faso, Kobo Collect was used for data collection. In-person face-to-face interviews were conducted for health workers, volunteers and caregivers to collect data on waiting time, local daily farming wage rate, etc. Additional cost data were collected from the three study countries during the full campaign season, from programme records at the National Malaria Control Program offices. At national, regional and district levels, SMC-related activities such as planning, monitoring and supervision, training, logistics distribution, data capture, pharmacovigilance, etc. were collected through in-person interviews and extraction from programme records. We also collected data for the total number of SMC doses administered in each community from programme records (online supplemental file 1).

10.1136/bmjgh-2024-018042.supp1 Supplementary data

### Data analysis

In this study, the financial cost of an intervention represents the amount of money that was paid for the resources being used. In contrast, economic costs represent the full value of the resources used in providing an intervention. Therefore, they aim to reflect the opportunity cost of resources, focusing on the value of the next-best alternative use that has been sacrificed as a result of using the resources, rather than just the monetary amount paid for them.^25^ Typical examples include costs such as unpaid volunteer distributors, caregivers’ time, etc.

To estimate economic costs, the resources used up in implementing each programme activity listed above were identified and measured in appropriate units. These include direct costs (recurrent costs such as medical supplies—SP+AQ—fuel for vehicle operation and maintenance, other non-medical supplies; and capital costs such as vehicles and equipment). The recurrent costs are those costs of items that have a useful life of less than 1 year. All capital items were identified to be those with useful lives of more than 1 year. Subsequently, all capital items were annualised using a standard discount rate—3%—and useful lives, to calculate annual economic costs of capital items. The useful life of vehicles, such as pick-up trucks; 4WD, for example, Toyota Landcruiser; and motorbikes used in SMC activities, was estimated to be 5 and 3 years, respectively.^24^ Similarly, an average useful lifespan of 3 years was assumed for office equipment, such as computers and printers. Furthermore, estimated national, regional and district costs associated with SMC such as planning, monitoring and supervision, social mobilisation, pharmacovigilance and training were allocated to the sampled communities according to number of doses administered in each community.

Indirect costs associated with the various activities included health worker, community volunteer and caregiver times contributed to the intervention, which were collected during surveys. We used national minimum wages per country^26^ to estimate health worker, volunteers and caregivers’ opportunity costs. The number of days worked on the SMC intervention by volunteers and health workers was obtained from district level and interviews with volunteers. To estimate the caregivers’ productivity losses, we collected data on the waiting time of caregivers during the first day of the SMC administration to the children. Subsequently, the study estimated households’ productivity losses for second and third doses by assuming that caregivers used half (50%) of the waiting time of the first day of SMC administration by distributors. The monetary value of workdays lost was calculated by multiplying the number of workdays lost by the prevailing local farming wage rate (a theoretical measure of labour productivity)^24^ in the communities.

In this study, ‘waiting time’ was operationalised as ‘the time period for which caregivers stayed home to wait for community distributors to visit the household to administer SMC medication to the child/children’.^24^

In estimating economic costs, we used the opportunity cost approach, which includes the financial costs of all resources expended (for market goods and services, these would often be using the market prices). In addition, we identified resources that come at no financial cost to programme implementers (eg, items donated by partners, the private sector, etc.), quantified them using standard costing approaches and included them. Again, we used the indirect cost approach described above to value the opportunity cost of resources from communities that are often not compensated for (eg, the time of community volunteers, opinion leaders and caregivers).

Financial cost and economic cost per monthly dose were estimated for the first and third cycles separately in each country. Subsequently, the annual cost for a child expected to receive all four doses was estimated by adding the cost per monthly dose for the first cycle to the average cost per dose of the third cycle × 3. Here, the assumption used was that the cost per monthly dose for the third cycle is likely to be identical for the second and fourth cycles. All costs are reported in 2022 US dollars at an exchange rate of CFA594=US$1.00.^27^ A one-way sensitivity analysis was conducted using the daily farming wage rate in each country to estimate the productivity losses of volunteers and caregivers.

## Results

### Cost per monthly dose ($) in Mali, Senegal and Burkina Faso

Table 1 shows the financial and economic costs per monthly dose in the three countries. In the first cycle, financial cost per dose ranged between US$1.15 and US$2.09. In Mali, where the financial cost per month was the lowest, the cost was US$1.15 (95% CI 1.00 to 1.30). In Burkina Faso, the cost was US$1.66 (95% CI 1.36 to 1.92), and Senegal recorded the highest financial cost per dose, US$2.09 (95% CI 1.88 to 2.29). In the third cycle, the financial cost was relatively lower in all the countries, ranging from US$0.94 (95% CI 0.80 to 1.07) in Mali to US$1.34 (95% CI 1.16 to 1.52) in Burkina Faso and US$1.32 (95% CI 1.20 to 1.44) in Senegal.

**Table 1:** Cost per monthly dose ($) in Mali, Senegal and Burkina Faso

Item	Mali	Senegal	Burkina Faso
Cycle 1			
Financial cost	1.15 (95% CI 1.00 to 1.30)	2.09 (95% CI 1.88 to 2.29)	1.66 (95% CI 1.36 to 1.92)
Economic cost	3.06 (95% CI 2.82 to 3.31)	3.70 (95% CI 3.50 to 3.90)	3.42 (95% CI 2.95 to 3.89)
Cycle 3			
Financial cost	0.94 (95% CI 0.80 to 1.07)	1.32 (95% CI 1.20 to 1.44)	1.34 (95% CI 1.16 to 1.52)
Economic cost	2.93 (95% CI 2.70 to 3.16)	2.79 (95% CI 2.66 to 2.92)	3.05 (95% CI 2.64 to 3.47)

Annual financial and economic costs per child expected to receive all four doses.

For economic cost per monthly dose, in the first cycle, Senegal recorded the highest cost of US$3.70 (95% CI 3.50 to 3.90) followed by Burkina Faso US$3.42 (95% CI 2.95 to 3.89) and Mali US$3.06 (95% CI 2.82 to 3.31). In the third cycle, the economic cost per monthly dose in all three countries was relatively lower than that of the first cycle (table 1). However, in the third cycle, the economic cost was highest in Burkina Faso US$3.05 (95% CI 2.64 to 3.47), followed by Mali US$2.93 (95% CI 2.70 to 3.16) and Senegal US$2.79 (95% CI 2.66 to 2.92).

**Figure 1:**
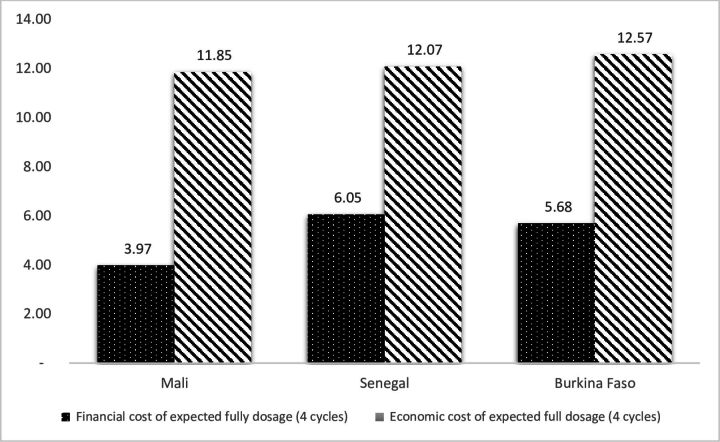
Annual financial and economic costs per child who received all four doses.

In figure 1, the annual financial and economic costs per fully dosed child are presented. The highest financial cost per child expected to receive all four doses was recorded in Senegal at US$6.05. In Burkina Faso and Mali, the estimated financial cost per child who received all four doses was US$5.68 and US$3.97, respectively. Annual economic cost per child reported to have received all four doses was estimated to be US$11.85 in Mali, US$12.07 in Senegal and US$12.57 in Burkina Faso (figure 1). In the context of Senegal, where three cycles are implemented in some areas, the annual financial cost and economic cost are US$4.54 and US$9.05 respectively. In Mali and Burkina Faso, some districts were implementing five cycles; as such, their annual financial costs are US$4.96 and US$7.10, while economic costs are US$14.81 and US$15.71, respectively.

### Breakdown of economic cost—direct and indirect costs

**Figure 2:**
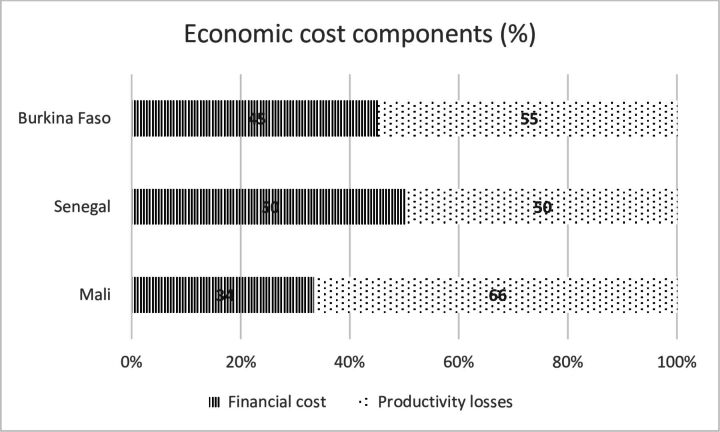
Breakdown of economic cost—direct and indirect costs.

Figure 2 shows distribution of direct and indirect costs for SMC cost in Mali, Burkina Faso and Senegal. The economic costs in all countries are dominated by indirect costs. The indirect cost ranged between 50% and 66% of the total economic cost. In Senegal, productivity losses were 50% of the total economic cost. In Burkina Faso, indirect cost constitutes 55% of the total economic cost incurred. In Mali, the productivity losses were approximately two-thirds (66%) of the total economic cost incurred on the SMC.

### Average economic cost of SMC activities in Mali, Senegal and Burkina Faso

In Table 2, the distribution of economic cost according to activities undertaken during the SMC implementation is presented. In all three countries, the most significant cost driver was caregiver cost, which is estimated to be approximately 17%–51% of average economic cost, with the highest recorded in Mali. The second most significant cost driver across all countries was SMC administration, constituting approximately 25%–31% of the total economic cost. District-level cost represents the average cost allocation per facility in the district constituting 9% of economic cost in Mali, 12% in Burkina Faso and 14% in Senegal (table 2). In Burkina Faso, training of volunteers and health workers, monitoring and supervision, and pharmacovigilance were 1.76%, 8.32% and 6.34% of economic cost, respectively. In Senegal, training of volunteers and health workers and monitoring and supervision were approximately 3.38% and 6.81% of economic cost, respectively, while pharmacovigilance was about 1%. In Mali, training and monitoring and supervision were 0.90% and 1.72%, respectively, while pharmacovigilance was less than 1%. Social mobilisation was a significant cost component in Senegal (4.70%) than in Mali (0.08%) and Burkina Faso (0.63%).

**Table 2:** Average economic cost of SMC activities per country

	Mali		Senegal		Burkina Faso	
Activity	Average ($)	Cost profile (%)	Average ($)	Cost profile (%)	Average ($)	Cost profile (%)
Training of volunteers						
Personnel (trainers)	27.26	0.07	846.82	2.13	64.87	0.37
Personnel (volunteers and relays)	231.29	0.57	104.13	0.26	160.27	0.92
Supplies and materials	39.83	0.10	302.97	0.76	47.74	0.27
Building/office space	42.09	0.10	46.02	0.12	32.55	0.19
Vehicle operating cost (eg, fuel, toll)	24.52	0.06	42.76	0.11	–	–
**Subtotal**	364.99	0.90	1342.70	3.38	305.43	1.76
Monitoring and supervision						
Personnel	429.27	1.06	2382.74	6.00	1291.88	7.43
Supplies and materials	223.65	0.55	206.19	0.52	87.45	0.50
Vehicle operating cost (eg, fuel, toll)	46	0.11	118.08	0.30	68.06	0.39
**Subtotal**	698.92	1.72	2707.00	6.81	1447.39	8.32
Pharmacovigilance						
Personnel	346.41	0.85	300.26	0.76	1078.87	6.20
Supplies and materials	5.23	0.01	63.43	0.16	24.44	0.14
**Subtotal**	351.64	0.86	363.68	0.92	1103.31	6.34
Planning						
Personnel	72.47	0.18	–		131.1	0.75
Building/office space	0.58	0.00	–		43.04	0.25
**Subtotal**	73.06	0.18	–	–	174.14	1.00
Social mobilisation						
Personnel	34.3	0.08	1479.47	3.72	46.8	0.27
Supplies and materials	–		387.83	0.98	63.33	0.36
**Subtotal**	34.3	0.08	1867.29	4.70	110.13	0.63
SMC administration						
Personnel (volunteers and relays)	4244.36	10.43	7939.36	19.98	3208.54	18.45
Supplies and materials	1745.92	4.29	1382.47	3.48	515.91	2.97
Medicine	4056.33	9.97	2988.22	7.52	1568.37	9.02
Vehicle operating cost (eg, fuel, toll)	123.95	0.30	–		–	
Donated items	5.47	0.01	–		–	
**Subtotal**	10 176.03	**25.02**	12 310.05	**30.98**	5292.82	**30.43**
Data capture				–		
Personnel	198.15	0.49	263.81	0.66	588.88	3.39
Supplies and materials	34.09	0.08	324.39	0.82	161.44	0.93
Building/office space (rental)	107.85	0.27	118.97	0.30	82.77	0.48
**Subtotal**	**340.09**	**0.84**	**707.17**	**1.78**	**833.09**	**4.79**
Capital cost		–				–
Equipment (laptop, telephone, powerbank)	23.73	0.06	631.64	1.59	88.99	0.51
**Subtotal**	**23.73**	**0.06**	**631.64**	**1.59**	**88.99**	**0.51**
Productivity loss_training	13.06	0.03	306.64	0.77	32.89	0.19
Productivity loss_monitoring and supervision	276.7	0.68	1665.34	4.19	289.42	1.66
Productivity loss_planning	271.38	0.67	–		519.38	2.99
Productivity loss_pharmacovigilance	89.34	0.22	46.53	0.12	59.69	0.34
Productivity loss_SMC admin (volunteers)	2562.27	**6.30**	4934.19	**12.42**	1026.93	5.90
Productivity loss_data capture	138.36	0.34	114.27	0.29	201.88	1.16
Productivity loss_caregivers	20 885.29	**51.34**	6749.91	**16.99**	3522.40	**20.25**
District cost	3829.23	**9.41**	5386.58	**13.56**	2051.51	**11.79**
Regional cost	302.28	0.74	228.15	0.57	63.31	0.36
National cost	124.89	0.31	77.01	0.19	116.15	0.67
Wastage cost	121.69	0.30	291.5	0.73	154.61	0.89
**Total economic cost**	40 677.25	**100.00**	39 729.65	**100.00**	17 393.47	**100.00**

SMC, seasonal malaria chemoprevention.

## Discussion

This study is the first multicountry study to assess detailed opportunity cost of SMC implementation in Africa. This study captured all the costs of the parties involved in SMC implementation, including caregivers, volunteers and healthcare providers. All previous studies except one single-country study by Nonvignon et al.^24^ aimed at assessing cost of SMC implementation did not comprehensively capture all relevant costs.^24^ For instance, some studies did not include the opportunity of caregivers whose children receive SMC doses. Moreover, the study by Gilmartin et al.^17^ did not measure capital costs such as Ministry of Health/non-governmental organizations (NGOs) building space and pharmacovigilance systems.^17^ These studies that exclude other perspectives often result in underestimation of the true opportunity cost of SMC implementation in various countries. Notwithstanding, these studies are still relevant based on the intended aim of the study. For example, if only the government’s financing and resource mobilisation is the focus, cost estimation can be limited to health system perspective of analysis.

The annual average per child financial cost in this study was US$3.98–US$6.05 over four monthly cycles. This is comparable to a previous study in seven countries implementing SMC of US$2.50–US$7.99.^17^ However, in comparison with the current study, a relatively lower financial cost per child expected to receive all four doses at US$2.92 was reported in Mali, and in Senegal, US$1.22 was reported.^15 16^ It is, however, noteworthy that in the Senegal study, only three monthly cycles of SMC doses were administered. On the other hand, a study in Ghana which reports the cost of SMC implementation piloted in the Upper West Region found a significantly higher financial cost of US$9.66 per fully dosed child for four cycles.^24^ The higher financial cost reported in Ghana may be explained by the pilot nature of the SMC implementation, which means that start-up cost was a key cost component.

We highlight the repeated cycle data collection that was used, which was not used in previous studies except Diawara *et al* 15 and Pitt *et al* 16 where the authors used a similar approach. This was important for the study to capture potential cost variations that may be associated with different cycles. As noted in our findings, this study found the first cycle costs marginally higher than the third cycle. For example, the financial cost per the first monthly cycle was US$1.15–US$2.09 compared with US$0.94–US$1.34 in the third cycle. Similarly, the economic cost was higher in the first monthly cycle (US$3.06–US$3.70) than in the third cycle (US$2.79–US$3.05). The cost variations in the cycles may be associated with relevant activities such as training and social mobilisation which are required to be undertaken more intensely in the first cycle but may be less intense or not performed in the third cycle based on prevailing circumstances. Moreover, even for instances where training may be undertaken, it may only be a refresher and not as intensive as training workshops towards the first cycle SMC administration.

Economic cost for children expected to receive all four doses was US$11.85–US$12.57. This cost is significantly lower than that in the study in Ghana, even though both studies measured the full opportunity cost of SMC implementation. In the Ghanaian study, Nonvignon *et al* 24 reported a cost (in 2015) of US$22.53 per fully dosed child.^24^ This translates into a US$5.63 per monthly dose which is higher than the US$2.96–US$3.14 reported in this study. Other studies have reported significantly lower economic cost per monthly dose (US$0.68–US$2.05) and four cycles of SMC (US$2.71–US$8.20 per child).^17^ Using 2010 cost data, Pitt and colleagues^16^ estimated US$0.38–US$2.74 of economic cost per child aged up to 10 years with one monthly dose of SMC.^16^ The economic cost of SMC per child fully adherent was US$6.38 in Mali.^15^ It is noteworthy that in both the studies in Mali and Senegal, cost evaluation was conducted from the health provider perspective. This is worth highlighting because this perspective is narrower compared with the societal perspective which includes all parties involved in the SMC implementation including volunteers and caregivers. The current study used the societal perspective and captured all opportunity costs to all parties. In addition, costs at national, regional and district levels were proportionately added to unit costs to ensure no opportunity cost is left out. These are some of the reasons why the current study has significantly higher economic cost than previous studies, except Nonvignon *et al*.^24^

This study shows that the economic cost was dominated by productivity losses, which accounted for 50%–66% of total economic cost. This large indirect cost is not surprising because of the frequency of SMC administration and the involvement of particularly volunteers and caregivers, who have to leave work and wait to receive volunteers to administer the drugs to their children. Interestingly, in the study by Pitt *et al.*,^16^ the authors argue that their choice of a health system perspective for the cost analysis is because the ‘opportunity cost for households to participate in SMC is expected to be low as SMC is delivered door-to-door’ as noted by Conteh *et al*.^16 28^ In contrast, however, the findings from this current study show that household opportunity cost is significant, and it is a major cost driver for all productivity losses incurred. This corroborates previous studies in Northern Ghana, where productivity losses accounted for 74% of total economic cost.^24^ It is noteworthy that a recent systematic review of studies that estimated the cost and cost-effectiveness of SMC implementation found that all the studies did not comprehensively assess cost from a societal perspective, except Nonvignon *et al* 24.^19^ It is therefore unsurprising that this study, which used the same approach, is recording comparable results. Measuring the full opportunity cost of SMC intervention has financial and economic implications for governments and funders. Moreover, the full opportunity cost of SMC implementation will make decision makers understand how to choose between equally effective malaria prevention interventions such as seasonal injectables and malaria vaccines. In essence, household and community resources represent significant community contributions to disease control and have implications on community and household incomes, particularly communities with large informal sectors.

Some limitations of the study are acknowledged. Foremost, this study did not assess the cost savings that society enjoys because of SMC implementation averting malaria cases. This is a benefit that can offset some of the opportunity cost of SMC implementation. Future studies can consider the cost savings accrued to society because of averted healthcare costs and out-of-pocket payments of caregivers, and also the longer-term benefits, for example, through better cognitive development, higher educational attainment and longer-term economic productivity.

Again, the study did not conduct data collection during four cycles, so it was assumed that two cycles captured average was sufficient. This limitation, notwithstanding the consideration of data from two cycles, is an improvement over previous study. Furthermore, some children may experience adverse reactions when they receive the medicine. This may cause their caregivers to incur out-of-pocket expenditure to treat. We did not capture this cost that caregivers may have incurred. In addition, this study did not capture fixed versus door-to-door costs, mainly because all the areas assessed used door-to-door approach, except for missed cases where caregivers sometimes have the opportunity to take them to a fixed point in a health facility. Furthermore, the study captured second and third doses administered by households in productivity losses. However, this assumed that caregivers used half (50%) of the waiting time of the first day of administration by volunteers. Regarding productivity losses incurred by caregivers, the study did not account for caregivers who take care of multiple children. This means that the productivity losses for caregivers with multiple children may have been overestimated. Finally, we acknowledge that we did not ascertain what caregivers were doing while waiting for volunteers to administer SMC to eligible children. The activity caregivers undertook during the waiting time can affect the cost of productivity losses reported. We therefore suggest a future qualitative study that will explore how caregivers spend their time while waiting in the house to receive SMC for their children.

## Conclusion

In conclusion, this study is the first to comprehensively estimate opportunity cost of multicountry SMC campaign targeting children 3–59 months in the Sahel region of sub-Saharan Africa and using repeated data collection. This study concludes that while SMC implementation comes with benefits to communities through reduced burden, it also leaves productive losses to households and volunteers, which is often ignored. Consequently, decision makers, including Ministries of Health and National Malaria Control and Elimination Programs in the sub-Saharan African region, should factor it in their resource allocation decisions and prioritisation of other effective malaria prevention interventions in children.

Moreover, we recommend the need for countries to routinely collect cost data. This will support rapid cost evaluation of malaria interventions and their economic implications to guide resource allocation decisions. Similarly, countries routinely collecting SMC coverage data will be critical to support SMC implementation decisions. Also, considering the higher indirect costs incurred by households, it is recommended that future studies conduct gender analysis to ascertain who is incurring the higher productivity losses between men and women. Finally, based on the findings, programme implementers should find a way to potentially reduce costs of SMC while maintaining coverage.

## Data Availability

All data relevant to the study are included in the article or uploaded as supplementary information.

## References

[1] WHO . WHO policy recommendation: Seasonal Malaria Chemoprevention (SMC) for plasmodium falciparum malaria control in highly seasonal transmission areas of the Sahel Sub-Region in Africa. Geneva: World Health Organization, 2012.

[2] WHO . WHO guidelines for Malaria. Geneva, 2023.

[3] Merle CS , Badiane NA , Affoukou CD , et al . Implementation strategies for the introduction of the RTS,S/AS01 (RTS,S) Malaria vaccine in countries with areas of highly seasonal transmission: workshop meeting report. Malar J 2023;22:242. 10.1186/s12936-023-04657-5 37612716 PMC10464391

[4] World Health Organization . World Malaria report 2024: addressing inequity in the Global Malaria response. Geneva: World Health Organization, 2024.

[5] Thwing J , Williamson J , Cavros I , et al . Systematic Review and Meta-Analysis of Seasonal Malaria Chemoprevention. Am J Trop Med Hyg 2024;110:20–31. 10.4269/ajtmh.23-0481 PMC10793029 38081050

[6] Baba E , Hamade P , Kivumbi H , et al . Effectiveness of seasonal malaria chemoprevention at scale in west and central Africa: an observational study. The Lancet 2020;396:1829–40. 10.1016/S0140-6736(20)32227-3 PMC7718580 33278936

[7] Konaté D , Diawara SI , Touré M , et al . Effect of routine seasonal malaria chemoprevention on malaria trends in children under 5 years in Dangassa, Mali. Malar J 2020;19:137. 10.1186/s12936-020-03202-y 32252774 PMC7137428

[8] Ambe JP , Balogun ST , Waziri MB , et al . Impacts of Seasonal Malaria Chemoprevention on Malaria Burden among under Five-Year-Old Children in Borno State, Nigeria. J Trop Med 2020;2020:9372457. 10.1155/2020/9372457 32665781 PMC7349624

[9] Tagbor H , Antwi GD , Acheampong PR , et al . Seasonal malaria chemoprevention in an area of extended seasonal transmission in Ashanti, Ghana: an individually randomised clinical trial. Trop Med Int Health 2016;21:224–35. 10.1111/tmi.12642 26578353 PMC4982104

[10] Cairns ME , Sagara I , Zongo I , et al . Evaluation of seasonal malaria chemoprevention in two areas of intense seasonal malaria transmission: Secondary analysis of a household-randomised, placebo-controlled trial in Houndé District, Burkina Faso and Bougouni District, Mali. PLoS Med 2020;17:e1003214. 10.1371/journal.pmed.1003214 32822362 PMC7442230

[11] Bakai TA , Thomas A , Iwaz J , et al . Effectiveness of seasonal malaria chemoprevention in three regions of Togo: a population-based longitudinal study from 2013 to 2020. Malar J 2022;21:400. 10.1186/s12936-022-04434-w 36587191 PMC9804945

[12] Cissé B , Ba EH , Sokhna C , et al . Effectiveness of Seasonal Malaria Chemoprevention in Children under Ten Years of Age in Senegal: A Stepped-Wedge Cluster-Randomised Trial. PLoS Med 2016;13:e1002175. 10.1371/journal.pmed.1002175 27875528 PMC5119693

[13] World Health Organization . Seasonal Malaria chemoprevention with sulfadoxine-pyrimethamine plus amodiaquine in children: a field guide. 2nd edn. 2023.

[14] OPT-smc: implementation research to optimize delivery and effectiveness of seasonal malaria chemoprevention 1 who technical consultation on seasonal malaria prevention: evidence review and data requirements for policy update. 2020.

[15] Diawara H , Walker P , Cairns M , et al . Cost-effectiveness of district-wide seasonal malaria chemoprevention when implemented through routine malaria control programme in Kita, Mali using fixed point distribution. Malar J 2021;20:128. 10.1186/s12936-021-03653-x 33663488 PMC7934250

[16] Pitt C , Ndiaye M , Conteh L , et al . Large-scale delivery of seasonal malaria chemoprevention to children under 10 in Senegal: an economic analysis. Health Policy Plan 2017;32:1256–66. 10.1093/heapol/czx084 28981665 PMC5886061

[17] Gilmartin C , Nonvignon J , Cairns M , et al . Seasonal malaria chemoprevention in the Sahel subregion of Africa: a cost-effectiveness and cost-savings analysis. Lancet Glob Health 2021;9:e199–208. 10.1016/S2214-109X(20)30475-7 33482140

[18] Faye S , Cico A , Gueye AB , et al . Scaling up malaria intervention “packages” in Senegal: using cost effectiveness data for improving allocative efficiency and programmatic decision-making. Malar J 2018;17:159. 10.1186/s12936-018-2305-6 29636051 PMC5894199

[19] Ruisch A , Iodice M , Mathur I , et al . Systematic review on the cost of seasonal malaria chemoprevention (SMC). Malar J 2024;23:384. 10.1186/s12936-024-05217-1 39695670 PMC11657606

[20] World Health Organization . WHO guidelines for Malaria. World Health Organization; 2024. Available: 10.2471/B09146

[21] WHO . Updated who recommendations for Malaria chemoprevention among children and pregnant women 2023. Available: https://www.who.int/news/item/03-06-2022-Updated-WHO-recommendations-for-malaria-chemoprevention-among-children-and-pregnant-women [Accessed 17 Dec 2023].

[22] World Health Organization . WHO guidelines for Malaria. 2022.

[23] Harvard University . Sample design optimizer. Available: https://immunizationeconomics.org/sample-design-optimizer [Accessed 23 Aug 2022].

[24] Nonvignon J , Aryeetey GC , Issah S , et al . Cost-effectiveness of seasonal malaria chemoprevention in upper west region of Ghana. Malar J 2016;15:367. 10.1186/s12936-016-1418-z 27423900 PMC4947302

[25] Turner HC , Sandmann FG , Downey LE , et al . What are economic costs and when should they be used in health economic studies? Cost Eff Resour Alloc 2023;21:31. 10.1186/s12962-023-00436-w 37189118 PMC10184080

[26] Minimum Wage . Minimum wage - mali - wageindicator.org. 2024. Available: https://wageindicator.org/salary/minimum-wage/mali [Accessed 22 Jul 2024].

[27] 1 usd to xof - US dollars to CFA Francs exchange rate. Available: https://www.xe.com/currencyconverter/convert/?Amount=1&From=USD&To=XOF [Accessed 22 Jul 2024].

[28] Conteh L , Patouillard E , Kweku M , et al . Cost effectiveness of seasonal intermittent preventive treatment using amodiaquine & artesunate or sulphadoxine-pyrimethamine in Ghanaian children. PLoS One 2010;5:e12223. 10.1371/journal.pone.0012223 20808923 PMC2923188

