# Supplementary material for: Estimating the opportunity cost of seasonal malaria chemoprevention implementation in Burkina Faso, Mali and Senegal
Source: BMJ Glob Health. 2025 Oct 3;10(10):e018042. doi: 10.1136/bmjgh-2024-018042 (PMC12551557; doi:10.1136/bmjgh-2024-018042)
Supplement: Supplementary data [file bmjgh-10-10-s001.pdf]

Number of SMC Doses per Community

**Mali**	**First cycle**	**Third cycle**
**Community**	**Number of doses**	**Number of doses**
Diema Central	7205	7205
Diancounte Camara	4825	4825
Koungo	3921	3921
Bema	4684	4684
Kouikoroba	12205	12205
Kolebougou	17067	17067
Souban	2501	2501
Tienfala	1963	1963
Yanfolila Central	10388	10388
Lontola	438	438
Soloba	1912	1912
Badogo	3709	3709
Sebougou	4993	4993
Secoura	7491	7491
Darsalam	12304	12304
Sinzana Gara	11017	11017
**Senegal**	**First cycle**	**Third cycle**
**Community**	**Number of doses**	**Number of doses**
Afia	4977	5158
Bandafassi	3900	3900
Botou	2800	3200
CPS DL	10600	10600
Dalaba	2082	2056
Dindefero	3500	3500
Dioulocon	2255	2255
Koussanar	6953	6953
Medina Cherif	2959	2959
Médinatoul	10000	10000
Patar	5725	5725
Saré Kemo	6900	6900
Tessan	2941	2941
Thietty	4977	4977
Walalane	3500	3500
**Burkina Faso**	**First cycle**	**Third cycle**
**Community**	**Number of doses**	**Number of doses**
CSPS Communal	5077	5077
CSPS de Bonyobo	2769	2769
CSPS de Iolonioro	3938	4119
CSPS de Lattou	678	688

Owusu R, *et al. BMJ Glob Health* 2025; 10:e018042. doi: 10.1136/bmjgh-2024-018042

CSPS de Niceo	1814	1077
CSPS de Oullo	720	720
CSPS de Secteur 10	1942	2428
CSPS de Secteur 4	4760	4760
CSPS de secteur 9	2371	3100
CSPS de Tiankoura	979	979
CSPS Kopoi	1051	1212
CSPS Palogo	1713	2000
CSPS Pouni Nord	2838	2838
CSPS Urbain1 boromo	4603	5217
CSPS Villy	2534	2534

Owusu R, *et al. BMJ Glob Health* 2025; 10:e018042. doi: 10.1136/bmjgh-2024-018042
